# VIRTUAL HEALTH COACHING TO IMPROVE HEART FAILURE CAREGIVERS’ SELF-CARE OUTCOMES

**DOI:** 10.1093/geroni/igae098.1512

**Published:** 2024-12-31

**Authors:** Karen Hirschman, Tracie Walser, Ryan Quinn, Barbara Riegel

**Affiliations:** University of Pennsylvania, Philadelphia, Pennsylvania, United States; University of Pennsylvania, Philadelphia, Pennsylvania, United States; University of Pennsylvania, Philadelphia, Pennsylvania, United States; University of Pennsylvania, Philadelphia, Pennsylvania, United States

## Abstract

Health coaching is assumed to improve self-care, but this assumption has not been well-tested. This randomized controlled trial involved 250 adults with health self-care neglect who provided >= 8 hours of care/week to their relative living with heart failure. We assessed the impact of virtual health coaching (n=125) on self-care compared to health information alone (n=125). The Virtual Health Coach for You (ViCCY) intervention included 10 synchronous sessions with a health coach over 6 months. ViCCY and health information were delivered via a computer tablet. Self-care data were collected with the Self-Care Inventory (SCI) (higher scores indicated better self-care.) at baseline and 6 months (August 2019-April 2023). Caregivers were primarily female (85%), white (62%), spouses (72%), and on average 55 years old (+/-14 years, 19-79). Almost half had a college degree (46%) and were employed full-time (44%). On average caregivers received 6 ViCCY sessions (median: 9) with 62% completing all 10 sessions, 28% completing 1-9 sessions, and 16% completing no sessions. The group-by-time interaction showed significant self-care improvement in the ViCCY group compared to the health information only (p=0.01). Self-care scores improved by 10 points indicating a clinically meaningful improvement at 6 months. Neither the number of health coaching sessions nor the total amount of time spent with the caregivers were statistically associated with the change in self-care rating over time in the VICCY group. The virtual health coaching intervention effectively improved self-care practices among heart failure caregivers, highlighting its potential to positively impact caregiver well-being and support.

